# Ecological acclimation of microbial communities to rising temperatures: growth, respiration, and interactions

**DOI:** 10.1128/aem.00218-26

**Published:** 2026-06-04

**Authors:** Wasilah Balogun, Mawada Elmhadi, He Fu

**Affiliations:** 1 Department of Biology, College of Science and Technology, North Carolina Agricultural and Technical State University 3616, Greensboro, North Carolina, USA; The Pennsylvania State University, University Park, Pennsylvania, USA

**Keywords:** microbial interactions, microbial growth, microbial respiration, carbon use efficiency, thermal response, climate change

## Abstract

Temperature is a fundamental regulator of microbial physiology, shaping processes from growth and respiration to community interactions and ecosystem functioning. This review synthesizes recent experimental and theoretical advances that reveal how microbes respond to warming across biological scales, with a focus on short-term ecological acclimations of microbial communities. At the cellular level, rising temperature affects enzyme kinetics, membrane fluidity, and metabolic efficiency, often in non-linear ways that challenge the validity of fixed *Q*_10_-based models. At the community level, warming tends to favor thermotolerant and slow-growing taxa, while reconfiguring microbial interaction networks by shifting balances between competition, cooperation, and syntrophy. These structural changes can reduce functional redundancy and stability, yet prolonged warming may also foster the emergence of cohesive, resilient community architectures. Overall, we emphasize the need for integrative mechanistic frameworks that link thermal physiology, carbon-use efficiency, and microbial interactions to improve predictions of microbial contributions to carbon cycling under climate change.

## INTRODUCTION

Temperature is a fundamental selective force for microbes, influencing enzyme activity, membrane dynamics, gene expression, and ultimately, growth and respiration. Recent syntheses posit that those thermal constraints shape not only individual physiology but also community assembly, coexistence, biogeography, and ecosystem functioning (1). Concurrently, trait-based theory connects thermal physiological variation to diversity and coexistence patterns, while integrated models of respiration and growth across temperatures provide a unified framework for microbial responses in biogeochemical models (2, 3).

Warming consistently alters microbial communities across ecosystems. In mountain streams, experimental warming shifted bacterial composition while maintaining broad functional profiles, indicating community turnover without functional loss (4). In a ~50-year subarctic soil warming experiment, higher temperatures enhanced growth by activating more taxa rather than individual taxa, revealing a compositional mechanism for warming-induced activity (5). In aquatic systems, microbial communities respond more rapidly to warming than to cooling, emphasizing the roles of thermal history and directional change on ecological timescales (6). Together, these findings show that temperature reshapes both rates and the accessibility of taxa across habitats (4–6).

Temperature also modulates microbial interaction networks. In a defined model microbiome, shifts of just 7°C–14°C altered interspecies interaction strengths and biofilm structure, revealing temperature sensitivity in community-intrinsic traits beyond growth alone (7). Thermal responses evolve as well: bacterial lineages adapted in multispecies settings show divergent thermal traits from those evolved in monoculture, cautioning against generalizing isolate behavior to communities (8). These findings link thermal physiology with network structure and highlight eco-evolutionary feedback in shaping emergent community function.

Despite this progress, we still lack an integrated, variance-aware framework to predict how temperature shapes microbial growth, respiration, and interactions under realistic thermal fluctuations and dynamic resource conditions. At the growth level, we lack general principles for scaling traits like lag time, maximum growth rate, and biomass yield across taxa (3). At the respiration level, emerging models remain weakly linked to carbon-use efficiency (CUE) and rarely resolve the partitioning of respiration into maintenance vs growth across varying thermal means and variances (2). Resource availability further complicates these dynamics. For instance, elevated substrate levels can enhance compensatory responses and reduce apparent temperature sensitivity, aligning with simplified “warmer-faster” assumptions under specific conditions.

**Fig 1:**
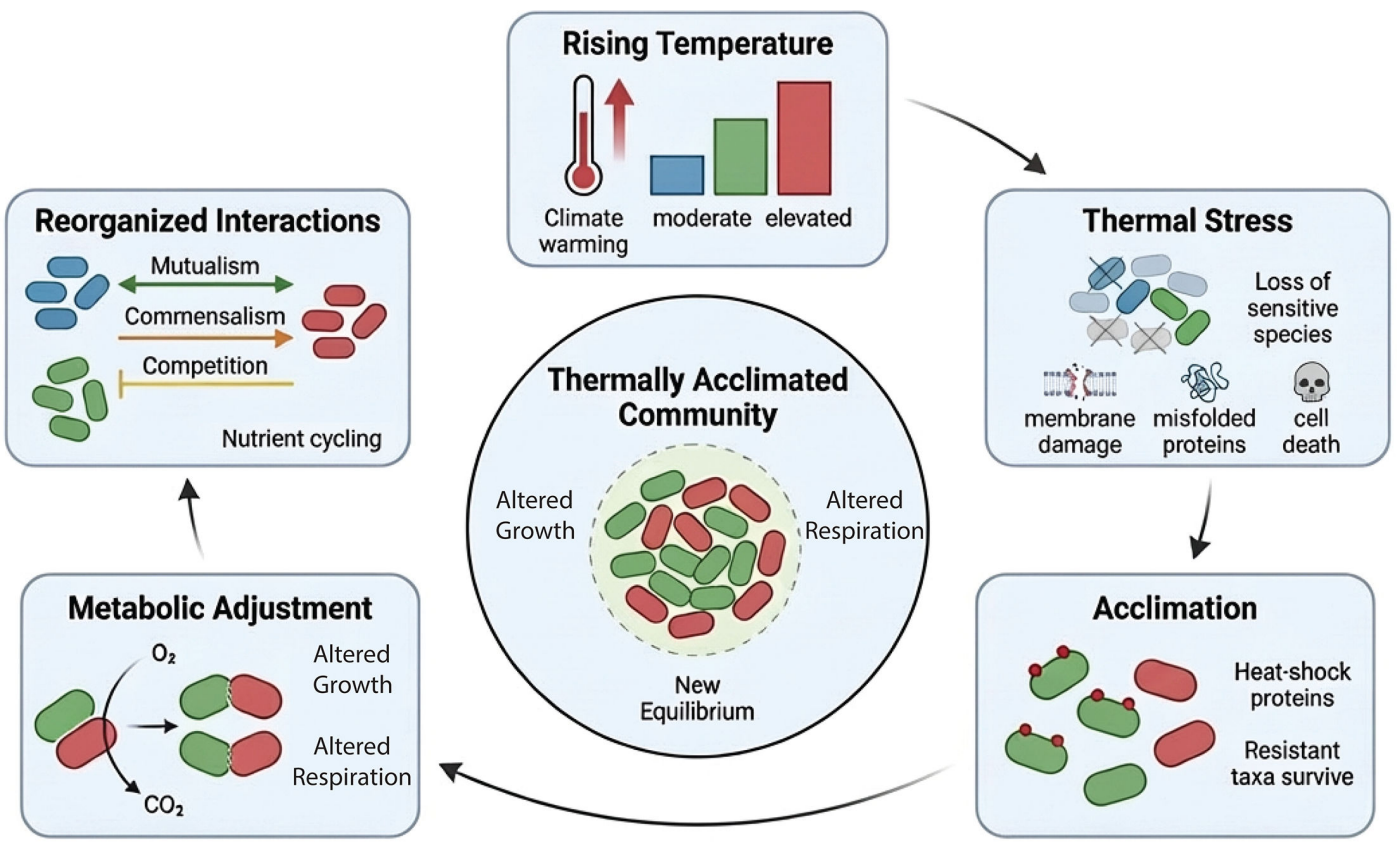
Ecological acclimation of microbial communities to warming. Increasing temperature modifies enzyme kinetics, membrane fluidity, and metabolic efficiency, leading to changes in growth and respiration while selecting for thermotolerant taxa and reshaping microbial interactions, including competition, cooperation, and mutualism.

In this review, we focus on short-term ecological acclimations, rather than evolutionary adaptations, of microbial communities to rising temperatures. We synthesize recent experimental and theoretical advances that reveal how microbial growth, respiration, and interactions respond to warming. We also discuss strategies and opportunities to address the knowledge gaps, primarily via integrating mechanistic frameworks that unite measurement, observation, experiment, and modeling efforts (Fig. 1).

## EFFECTS OF TEMPERATURE ON MICROBIAL GROWTH

### Temperature-dependent growth dynamics

#### Impacts of temperature on growth rate, lag phase, and biomass formation

Microbial growth rates are intrinsically linked to temperature, primarily due to temperature-sensitive enzyme activities that drive metabolic rearrangements (9, 10). Most microbes exhibit a well-defined thermal performance curve, characterized by an optimum temperature for maximal growth, flanked by minimum and maximum limits for survival and replication (11). For instance, species like *Pseudomonas aeruginosa* show clear growth impairment when temperatures exceed their optimal range (9, 10). This principle has broader applications, as biomass yields for certain microbial processes peak at moderate temperatures (e.g., ~25°C) and decline significantly at elevated ranges (e.g., 30°C–45°C) (12).

Beyond its effect on microbial multiplication, temperature also critically influences the lag phase, the period of physiological adjustment before active growth begins. This phase is highly temperature sensitive and has been quantified across species, such as *Geotrichum candidum*, between 6°C and 25°C (11). Rising temperatures typically increase growth rate and shorten lag duration, with a strong inverse correlation (13). Lag time may correlate with, or vary independently from, maximum growth rate, influenced by pH, temperature, and osmolarity (14). Multistep models highlight the role of both temperature and culture history, for example, higher preculture temperatures shorten lag in psychrotrophs and *Listeria monocytogenes* (15, 16). These insights into lag phase are pivotal for determining and understanding microbial community dynamics under variable environments and resilience to temperature shifts.

Ultimately, the combined effects of temperature on lag phase and growth rate shape overall microbial and ecosystem biomass yield. Temperature strongly influences biomass production across scales, from microbes to global ecosystems. In biogas systems, it affects algal growth, with strains like *Chlorella vulgaris* studied under varying mesophilic conditions (12). In terrestrial ecosystems, warming alters aboveground biomass, with optimal temperatures for rapid growth (~22.3°C) differing from those for maximal biomass (~16.4°C) in old-growth rainforests (17). At the microbial scale, warming often depletes biomass via carbon loss, while cooler periods support carbon storage (18). Together, these findings show temperature’s broad impact on biomass, with major implications for ecosystem resilience and resource management.

#### Optimal vs extreme temperature conditions and the viability of microbes

Microbial viability is strongly shaped by temperature. Within the optimal range, microorganisms achieve their highest rates of metabolism and growth. At the same time, enzyme activity remains stable, membranes function properly, and nutrient uptake is efficient, all of which support both natural environmental processes and industrial applications. For instance, fermentation relies on the maintenance of species-specific thermal optima to provide consistent metabolite yields, product quality, and microbial stability. Likewise, in engineered microbial systems such as encapsulated formulations, maintenance of proper thermal conditions preserves cellular viability during storage and application. Temperature stability forms the foundation for continuous microbial activity in marine and terrestrial environments, driving primary functions like nutrient cycling and community interactions that sustain larger ecological networks (1, 19).

Thermal extremes have an impact on microbial functions beyond population dynamics. Proteins and enzymes are denatured; bioactive compounds are decomposed; and cell membrane perturbation is induced (20). These effects can be detrimental to microbial functions in bioremediation, fermentation, or host interaction. In agricultural systems, an exceeded thermal tolerance of plants and their associated microbial communities can undermine crop yields by disrupting beneficial symbioses (21). On a broader scale, increased ambient temperatures have been associated with increased levels of foodborne disease, highlighting the public health implications of temperature shifts for microbial pathogens.

### Physiological responses to rising temperatures

Microorganisms have impressive short-term acclimation mechanisms capable of rapid physiological adaptation to temperature fluctuation. Such reactions are primarily regulated by modulation of enzyme activity and membrane structure reorganization, which together preserve cellular integrity and maintain metabolic homeostasis.

At the enzymatic level, rising temperatures trigger immediate biochemical rearrangement, particularly through the activation of antioxidant defense systems and heat-shock protein networks. Sublethal heat treatment of *Tetragenococcus halophilus* caused cross-protection against ethanol stress through the increased activities of catalase, superoxide dismutase, and chaperones, including GroEL and DnaK (22). Similarly, heat-induced thermotolerance in various groups of microorganisms is associated with the regulation of metabolic enzymes involved in energy generation and redox balance (23). These rapid enzymatic adjustments shield cells from oxidative stress and denaturation of proteins, maintaining catalytic efficiency at elevated temperatures.

Besides enzymatic regulation, membrane lipid remodeling represents another key mechanism of short-term physiological acclimation to temperature change. After 7 days of growth across a temperature gradient of 5°C, 15°C, 25°C, and 35°C, cold-tolerant bacteria showed pronounced shifts in fatty acid composition: gram-negative species shifted from monounsaturated toward saturated fatty acids, whereas gram-positive species increased their overall abundance of saturated fatty acids, including both branched- and straight-chain forms (24). A similar pattern has been observed in the thermophile *Thermus aquaticus*: when transferred from 50°C to 80°C, its fatty acid profile shifted toward greater synthesis of branched C_17_ fatty acids and reduced production of C_14_ fatty acids (25). Together, these lipid rearrangements likely act in concert with enzymatic adjustments to provide a rapid physiological buffer against thermal fluctuations.

## EFFECTS OF TEMPERATURE ON MICROBIAL RESPIRATION

### Temperature influences metabolic activity

#### Modifications in oxygen uptake, CO_2_ production, and ATP synthesis

Temperature affects enzymatic kinetics, membrane fluidity, and cellular energy balance, which is associated with oxygen consumption, carbon dioxide (CO_2_) evolution, and adenosine triphosphate (ATP) production that are central to microbial metabolism. Such interconnected processes govern the way microorganisms balance carbon cycling with energy generation. Short-term physiological acclimation processes permit microbial systems to maintain respiration and energy production within thermal gradients (26).

Aerobic metabolic capacity is defined by the oxygen uptake rate, which underpins microbial growth and survival. As temperatures rise, oxygen diffusion and biochemical demand become misaligned, constraining aerobic activity. Metabolic scaling models show that oxygen demand increases faster than supply with warming, narrowing the aerobic window for active metabolism (27). This reflects a peak in oxygen uptake efficiency at higher temperatures, beyond which diffusional limits prevent further metabolic increases despite continued thermal stimulation. The effect is especially pronounced in larger microbial cells and aquatic ectotherms, where diffusion-limited oxygen transfer restricts aerobic scope under warming (27).

Temperature-driven changes in CO_2_ production closely track shifts in microbial respiratory activity. Soil communities typically show increased respiration with rising temperatures, but this response plateaus as microbes reach peak metabolic efficiency. This compensatory adaptation helps prevent excessive CO_2_ loss under prolonged warming, reflecting thermal homeostasis in soil microbial populations. The extent of this response is tightly regulated by substrate availability: respiration is more temperature sensitive in carbon-rich soils but less so in carbon-poor ones. Moreover, empirical evidence suggests that substrate quality, rather than thermal acclimation alone, exerts the strongest influence on respiration rates (28). This implies that microbial metabolism is governed more by substrate-regulated enzyme activity than by temperature acclimation, underscoring the importance of integrating resource chemistry with temperature physiology in modeling CO_2_ flux.

At the cellular level, temperature regulates ATP production by modulating the proton motive force, membrane viscosity, and the kinetic efficiency of ATP synthase. At low temperatures, psychrophilic organisms exhibit compensatory adaptations that sustain energy yield despite reduced enzymatic activity. For example, the cold-adapted marine bacterium *Pseudoalteromonas haloplanktis* maintains ATP production by rerouting metabolic fluxes between major carbon pathways under suboptimal conditions (29). Similarly, psychrotrophic microbes like *Poseidonibacter antarcticus* enhance ATP synthesis and amino acid uptake at low temperatures by upregulating both substrate-level and oxidative phosphorylation (30). These findings highlight distinct energetic responses to thermal stress: psychrophiles maintain function by increasing ATP output, while thermophiles improve efficiency by minimizing maintenance costs.

From a thermodynamic perspective, the coupling of oxygen use, CO_2_ production, and ATP synthesis reflects universal energetic principles governing the temperature dependence of biological reactions. Early studies showed that microbial growth, survival, and maintenance share a common activation energy, suggesting that metabolic thermal sensitivity stems from fundamental biochemical kinetics (26). Combined with oxygen-limitation models, this framework suggests that rising temperatures accelerate enzymatic reactions but simultaneously reduce oxygen availability. The resulting imbalance shifts microbial energy investment away from biomass production toward maintenance and stress responses.

Together, temperature governs microbial metabolism through interlinked shifts in oxygen use, CO_2_ fixation, and ATP production. These processes are shaped by both kinetic constraints and regulatory mechanisms that enable microorganisms to optimize energy production and use under fluctuating thermal conditions. Ultimately, the interplay among substrate availability, thermodynamic efficiency, and oxygen limitation defines microbial roles in respiration, energy transfer, and carbon cycling within a warming biosphere.

#### *Q*_10_ coefficient and increased metabolic rate following warming

Biochemical and microbial process rates generally rise with temperature, often quantified by the *Q*_10_ coefficient, the ratio of rate increase for every 10°C temperature rise. While a *Q*_10_ value between 2 and 3 has traditionally represented biological temperature sensitivity, assuming a constant *Q*_10_ across environments oversimplifies the complex biochemical and ecological regulation of microbial metabolism. Empirical and modeling studies increasingly show that metabolic temperature dependence is non-linear, shaped by enzyme kinetics, substrate availability, and physiological adaptation rather than a fixed exponential function (29, 31).

At the biochemical level, enzyme-driven microbial growth and respiration follow Arrhenius-type dynamics, with reaction rates governed by activation energy. However, because microbial metabolism involves cascades of enzymatic reactions and physiological trade-offs, a single *Q*_10_ cannot adequately represent system-wide temperature sensitivity. Modeling evidence shows that *Q*_10_ values tend to decline at higher temperatures, reflecting lower activation barriers and increasing diffusion constraints (31). As a result, applying a uniform *Q*_10_ risks underestimating decomposition rates in cold soils and overestimating them in warmer ones, introducing significant bias into ecosystem-level carbon flux predictions.

Thermodynamically, temperature sensitivity is better described by the macromolecular rate theory (MMRT), which captures the unimodal nature of enzyme responses to changes in protein heat capacity and denaturation thresholds. MMRT predicts higher *Q*_10_ values at low temperatures—where activation energy dominates—and progressively lower values as enzymes approach their thermal optima, a pattern consistently observed across microbial systems (32). This mechanistic framework links molecular-level enzyme behavior to broader ecosystem metabolism, explaining why microbes in soil and aquatic environments often exhibit stronger temperature responses in colder settings and attenuated or declining sensitivity in warmer ones.

Beyond molecular kinetics, ecological context and resource availability significantly influence metabolic responses to warming. The rise in microbial respiration with temperature can be either amplified or dampened, depending on substrate supply, which fuels enzymatic activity. In carbon-rich conditions, microbes frequently display compensatory thermal adaptation, lowering their *Q*_10_ and maintaining metabolic efficiency at elevated temperatures (33). Under substrate limitation, however, *Q*_10_ increases as organisms struggle to meet energetic demands without sufficient resource reallocation. This plasticity highlights that warming-driven changes in microbial metabolism are not purely biochemical but emerge from interactive feedback between physiological flexibility and resource constraints.

Recent findings underscore the role of eco-evolutionary processes in modifying *Q*_10_ behavior over time. With continued warming, microbial communities can shift carbon investment toward producing extracellular enzymes, sustaining high decomposition rates even after thermal acclimation. This adaptive enzyme reallocation enhances soil carbon turnover, and long-term warming may drive emergent increases in metabolic activity, even when short-term *Q*_10_ responses appear subdued. Moreover, this metabolic acceleration extends to nitrogen cycling, where warming disrupts the balance between nitrification and mineralization, further enhancing microbial turnover and enzymatic activity (34). These coupled carbon–nitrogen dynamics reveal that warming influences microbial metabolism at both the biochemical and ecosystem nutrient levels.

Collectively, these insights suggest that the *Q*_10_ coefficient is not a fixed biological constant but rather an emergent property shaped by thermodynamic constraints, substrate feedback, and adaptive responses. While initial warming stimulates microbial metabolism by accelerating enzymatic reactions, long-term exposure induces acclimation and evolutionary compensation, moderating the intensity of response. Thus, integrating mechanistic models like MMRT with ecological and evolutionary frameworks provides a more accurate and dynamic basis for forecasting the impacts of global warming on microbial metabolism and soil carbon cycling.

### Temperature shifts efficiency of microbial carbon cycling and nutrient processing

Rising temperatures affect microbial carbon use efficiency (CUE)—the proportion of assimilated carbon allocated to biomass vs lost as CO_2_—thereby influencing the equilibrium between soil carbon storage and nutrient cycling. As an integrator of microbial physiology and ecosystem carbon fluxes, CUE links cellular metabolic responses to broader patterns of carbon retention and turnover in soils. Warming modifies microbial efficiency both directly, through temperature effects on enzyme kinetics, and indirectly, via changes in substrate availability, nutrient limitation, and eco-evolutionary adaptations.

At the physiological level, temperature alters the balance between microbial growth and maintenance, affecting how efficiently microbes recycle carbon into biomass. Evidence suggests that CUE’s temperature sensitivity varies with microbial growth strategy: fast-growing taxa tend to show declining CUE with rising temperatures, while slow-growing groups maintain higher efficiency under the same conditions (35). This pattern reflects a fundamental rate–yield trade-off: higher metabolic rates under warming elevate maintenance costs and respiration, reducing the proportion of carbon retained in biomass. These individual-level constraints scale up to influence the fate of soil organic carbon (SOC).

At the ecosystem scale, CUE–SOC relationships under warming are complex and context dependent. For instance, the direction of this relationship was shown to hinge on how temperature alters CUE itself: where CUE decreases with warming, SOC increases with rising CUE; where CUE increases, the trend reverses (36). This supports the conclusion that microbial efficiency under warming dictates whether soils function as carbon sources or sinks.

Seasonal dynamics further complicate this relationship. Asymmetric winter warming—where winter temperatures rise more than summer—leads to stronger reductions in microbial growth and CUE compared to year-round warming. This is attributed to increased carbon limitations and nutrient stress in subsequent growing seasons. Such seasonal effects suggest that models assuming uniform warming may underestimate soil carbon loss and nutrient imbalances.

Nutrient availability also modulates CUE responses to warming. Although nitrogen additions have limited direct effects on CUE (37), warming-induced nutrient depletion can suppress microbial efficiency indirectly by exacerbating carbon–nutrient stoichiometric imbalances. In such conditions, microbes divert more carbon to nutrient acquisition rather than biomass formation, thereby reducing CUE and altering nutrient turnover dynamics.

## EFFECTS OF TEMPERATURE ON MICROBIAL INTERACTIONS

### Community stability and species composition

Temperature exerts a profound influence on microbial communities, reshaping both their composition and diversity. Elevated thermal conditions often lead to reduced diversity and increased community dominance while simultaneously accelerating microbial processes such as respiration and carbon use efficiency (38). Studies from geothermal gradients indicate that microbial assemblages remain compositionally stable up to a certain thermal threshold, but undergo abrupt shifts once temperatures exceed critical thresholds of ~6°C to 9°C, suggesting a tipping point in community resilience (39). Moderate warming can initially boost diversity, yet sustained temperature increases tend to reduce it, as thermotolerant taxa such as *Bacillus* become dominant, marking a transition from coexistence to thermal filtering (40).

In aquatic systems, temperature similarly shapes microbial structures. For instance, in mountain stream sediments, phyla such as *Bacteroidota* shift markedly between freezing and non-freezing seasons, underscoring the role of thermal fluctuation in organizing sedimentary microbial networks (4). In marine environments, rising ocean temperatures favor slow-growing, metabolically conservative taxa, reflecting a community-wide adjustment toward thermal optimization in growth strategy.

Across terrestrial and aquatic systems, microbial diversity frequently follows a bell-shaped relationship with temperature, peaking at intermediate thermal ranges and declining under more extreme conditions (41). Functional and taxonomic assessments reveal that high temperatures select for microbes with smaller genomes and reduced functional specialization, contributing to lowered species richness and diminished functional redundancy (41). This reduction in redundancy weakens the capacity for ecosystem functional compensation under further warming. Arctic soil warming experiments support these findings, showing that prolonged exposure to elevated temperatures leads to reduced evenness and dominance by thermotolerant groups such as *Actinobacteria* (42).

The stability of microbial assemblages is also shaped by the rate and duration of temperature change. Under continuous warming, bacterial communities tend to become less connected and more vulnerable, whereas episodic heating may enhance resilience by selecting thermotolerant taxa capable of rapid recovery (41). These findings highlight that both the timing and magnitude of thermal exposure critically determine whether microbial communities adapt, reorganize, or collapse under warming pressures.

Thermal shifts also alter the balance between generalist and specialist species. In thermally stressed mangrove environments, specialists such as Alphaproteobacteria and Gammaproteobacteria dominate under high temperatures, while generalists persist under more moderate conditions. This pattern reflects a transition from functionally diverse, flexible communities to deterministic, niche-constrained structures with reduced ecological plasticity (43).

Together, the evidence suggests that warming selectively favors thermotolerant and specialized microbes, often at the cost of community diversity, evenness, and functional redundancy. While mild warming may temporarily boost diversity and metabolic potential, extreme or prolonged warming tends to erode community complexity and stability. The resulting loss of redundancy undermines the adaptive capacity of microbial ecosystems, creating more homogeneous but less resilient communities in the face of ongoing climate change.

### Changes in microbial interactions

#### Effects on keystone taxa and stability of microbial networks

Warmer temperatures reshape patterns of microbial stability by altering which species function as keystone taxa, thereby transforming the structure of microbial interaction networks. In some ecosystems, prolonged warming leads to increased network complexity and connectivity, with the emergence of new keystone taxa. This suggests that certain microbial communities adapt by forming tighter cooperative clusters that enhance resilience under climate warming (44). Such structural reinforcement often arises from slow-growing, resource-efficient K-strategists, which become central in reorganized networks. Particularly in ecosystems experiencing decadal warming, the restructuring of interactions, rather than changes in species richness alone, moderates carbon-cycle feedback and thermal adaptation of metabolic functions (45).

However, warming does not universally enhance network stability. In cold-region soils, such as permafrost ecosystems, elevated temperatures or thawing frequently lead to weakened network integration, reduced redundancy, and loss of keystone taxa, making microbial communities more vulnerable to disturbance and accelerating carbon loss (46). Experimental incubations confirm that warming often results in simplified microbial networks, with lower connectance and diminished positive interactions, ultimately eroding the influence of keystone taxa and increasing community sensitivity to environmental change (47).

Functional group identity also shapes microbial stability responses. Fungal networks tend to maintain higher complexity and robustness under warming conditions, often retaining their keystone roles even as bacterial networks become more fragile, indicating that fungi may act as stabilizing backbones in thermally stressed microbiomes (48). In contrast, studies on synthetic microbial communities have shown that warming-induced shifts in carrying capacity and interaction strength can suppress the emergence of keystone taxa altogether. In these systems, the presence of hierarchical and redundant structures limits the disproportionate influence of any single taxon (49).

Collectively, these findings underscore that microbial network stability under warming varies with thermal intensity, ecosystem context, and the dominant functional groups involved. While some communities reorganize adaptively through tighter cooperation and new keystone taxa, others experience simplification and destabilization, with important implications for biogeochemical cycling and ecosystem resilience.

#### Shifts in competition, cooperation, and syntrophy with warming

Warming reshapes microbial interaction networks by intensifying competitive pressures and weakening cooperative and syntrophic relationships that support ecosystem functioning. As metabolic rates increase with temperature, resource consumption accelerates, promoting a shift toward negative interaction links and simplified network architecture. Soil communities exposed to higher temperature exhibit reduced connectance and a higher proportion of antagonistic interactions, signaling a transition to competition-driven structuring under thermally demanding conditions (47). Similar patterns have been observed in plant–microbe interaction networks, where initial warming enhances connectivity through generalist facilitation, but continued temperature rise ultimately destabilizes microbial co-occurrence networks by selectively suppressing cooperative taxa (40).

Changes in network structure also shape community-level responses to warming. Facilitative communities can be more thermally responsive at first because cross-feeding and metabolic complementarity raise biomass production and strengthen the temperature dependence of respiration. Experimental work showed that shifting microbial assemblages toward facilitation amplified the thermal sensitivity of community respiration (50). Yet this effect often weakens under sustained warming. Field evidence indicates that warming can reduce network complexity and weaken the link between microbial associations and ecosystem functioning, as shown in alpine grasslands where warming decoupled microbial network complexity from ecosystem multifunctionality (51). Related work in rhizosphere soils also shows that stronger positive microbial associations are linked to higher carbon use efficiency, implying that disruption of cooperative ties may shift communities away from growth and toward maintenance respiration (52). Together, these studies show that warming does not simply speed metabolism through *Q*_10_ effects but also reorganizes microbial interactions in ways that determine whether communities translate higher temperature into greater biomass production, higher respiration, or lower functional stability.

Syntrophic and mutualistic associations appear particularly vulnerable to warming-induced restructuring. In anaerobic digestion systems, rising temperatures favor hydrogenotrophic methanogens over acetoclastic partners, fundamentally altering carbon-processing consortia (53). In marine systems, mutualistic interactions—such as heterotrophs protecting *Synechococcus* by scavenging reactive oxygen species—can be compromised when symbiotic partners exhibit mismatched thermal optima, disrupting essential autotroph–heterotroph dynamics (54). Theoretical work further suggests that warming reduces the selective advantage of cooperative thermogenesis, driving microbial communities toward traits favoring competitive dominance rather than shared buffering of thermal stress (55). Together, these findings indicate that warming shifts microbial communities from cooperative, functionally resilient networks toward more competitive and less stable configurations.

## LOOKING FORWARD: OPPORTUNITIES AND STRATEGIES

Recent work has begun to address remaining challenges in predicting microbial responses to variable thermal environments through trait-based theory, improved physiological models, and more realistic experimental designs. At the level of growth, trait-based frameworks are increasingly used to identify common thermal scaling relationships for traits such as lag time, maximum growth rate, and biomass yield across taxa (3). At the level of respiration, newer models are strengthening links with carbon-use efficiency and helping resolve the relative contributions of maintenance, growth, and respiration across different thermal means and variances (2). Meanwhile, new theory is beginning to link temperature and resource supply in a single framework, instead of treating them as separate drivers (56); new methods aim to infer both processes simultaneously from the same data stream, reducing scale mismatch and making it easier to separate carbon allocated to biomass production from carbon lost through respiration (57). These studies also highlight the importance of substrate availability in mediating thermal responses, including its ability to reduce apparent temperature sensitivity and, in some cases, produce dynamics consistent with simplified warmer-faster expectations.

More broadly, mechanistic models are increasingly combined with data-driven methods to improve predictive power. Hybrid frameworks that integrate deterministic models with machine learning can capture non-linear behavior while preserving biological interpretability. These approaches have improved predictions of microbial growth under fluctuating temperature and pH and show strong potential for real-world applications (58). At the same time, advances in parameter identifiability and sensitivity analysis are improving the reliability of complex models, particularly for microbial communities (59). Together, these efforts mark progress toward a more predictive understanding of microbial responses to warming.

Future progress will depend on stronger integration of growth, respiration, and species interactions across biological scales. Linking intracellular processes, population dynamics, and community interactions with high-throughput omics, improved numerical methods, and growing computational power should enable more detailed and predictive models. As envisioned previously, modeling efforts would ideally occur in collaboration with empirical investigations, where model scenarios and data acquisition inform each other (60). Such models could make important contributions to microbial ecology and global change biology.
